# Involvement of the central hypothalamic-pituitary-adrenal axis in hair growth and melanogenesis among different mouse strains

**DOI:** 10.1371/journal.pone.0202955

**Published:** 2018-10-24

**Authors:** Qian Wang, Huali Wu, Jia Zhou, Siran Pei, Jing Li, Yuanyuan Cai, Jing Shang

**Affiliations:** 1 State Key Laboratory of Natural Medicines, China Pharmaceutical University, Nanjing, Jiangsu, China; 2 Jiangsu Key Laboratory of TCM Evaluation and Translational Research, China Pharmaceutical University, Nanjing, Jiangsu, China; 3 Qinghai Key Laboratory of Tibetan Medicine Pharmacology and Safety Evaluation, Northwest Institute of Plateau Biology, Chinese Academy of Sciences, Xining, Qinghai, China; China Agricultural University, CHINA

## Abstract

Stress has been demonstrated to play an important role in hair follicle function and the pathogenesis of some hair disorders. The central hypothalamic-pituitary-adrenal (HPA) axis is activated by stress stimuli, synthesizes and releases various components and eventually induces the pathogenesis and recurrence of peripheral diseases. Our aim is to compare the different responses under exposure of stress in hair follicle function among different mouse strains, and to detect the involvement of the central HPA axis after stress in hair follicle growth and melanogenesis. In this study, we exposed different mouse strains (C57BL/6, CBA/J, C3H/HeN, BALB/c and ICR) to a 21-day chronic restraint stress protocol and selected C57BL/6, CBA/J and BALB/c mice for further study because of their significant behavioral alterations. Then, we evaluated and compared the different responses and sensitivity to chronic restraint stress in hair follicle function and central HPA axis among the selected strains. The results showed that expression of POMC, CRF and GR mRNA and protein and serum levels of corticosterone were inhibited in response to stress. These findings suggested that chronic restraint stress may inhibit hair follicle growth and melanogenesis via regulating the key elements of the central HPA axis. In addition, the results revealed different mouse strains exhibit different responses in the central HPA axis and hair follicle after stress exposure. C57BL/6 might be the most sensitive strain among the three strains tested as well as an appropriate strain to study possible pathophysiological mechanisms by which the nervous system influences skin function and screen dermatological drugs suitable for psychotherapy. We believe the current study will provide some useful information for researchers who are interested in the bidirectional communication between the nervous and skin systems and the management of stress-induced cutaneous diseases.

## Introduction

Psychosocial stress has been demonstrated to play an important role in hair follicle function and the pathogenesis of some hair disorders [1, 2]. According to case reports and epidemiological surveys, there is a 30% prevalence of psychiatric stress in dermatologic patients [3]. Vitiligo and hair loss are typical skin diseases with psychiatric symptoms [4, 5].

Arck *et al*. proposed a “brain-skin connection” theory to describe a link between stress and skin disorders. In brief, stress exposure leads to activation of the HPA axis and sympathetic system and exerts its effects on skin function through local neuronal plasticity and neuro-endocrine-immune interaction. Skin and brain share several same neurohormones, neurotransmitters, and neuropeptides, and these signals mediates and modulates systemic stress responses to challenge the skin’s cell homeostasis [6]. The responses to stress in the skin system are revealed as the typical effects on hair follicle growth and melanogenesis [7, 8]. Studies have reported that foot and sonic stress can induce hair follicle inhibition in C57BL/6 mice, including a decrease in hair follicle numbers, morphologic degeneration, changes in the hair cycle and overexpression of substance P (SP), calcitonin gene-related peptide (CGRP), and growth-associated protein-43 (Gap-43)-positive nerve fibers [9, 10]. Moreover, other stimulatory factors such as ultraviolet rays, neurohormones, and neuropeptides may affect melanocytes function in the neuroendocrine stress response [8]. Pang *et al*. found chronic restraint stress can suppress melanogenesis in C57BL/6 mice and mRNA expression levels of key factors in the cutaneous hypothalamic-pituitary-adrenal (HPA) axis [11]. Therefore, hair follicles offer an excellent model for us to study how psychological stress exerts it effects and regulates diseases.

Recent research has focused on the skin HPA axis, whereas there are few studies investigating the modulation of the central HPA axis on hair follicle function [12–14]. The systematic neuroendocrine pathways and mechanisms are still not clear. In the process of HPA axis activation, hypothalamic corticotropin-releasing hormone (CRH) is synthesized and secreted, inducing proopiomelanocortin (POMC) expression in the anterior pituitary gland. POMC is processed to POMC-derived hormones, such as adrenocorticotropic hormone (ACTH) and α-melanocyte-stimulating hormone (α-MSH). Pituitary ACTH binds to melanocortin (MC) type 2 receptors in the adrenal cortex and stimulates the synthesis and secretion of adrenal glucocorticoids (cortisol and corticosterone) into systemic circulation [15, 16]. In the hippocampus, glucocorticoids bind to glucocorticoid receptors (GRs), participating in the negative feedback regulation of the HPA axis through inhibiting the release of CRH and POMC [17, 18].

Published research has identified that mice and rats may regulate their stress systems differently due to their different genetic backgrounds [19–21]. These strain-dependent differences can serve as a powerful tool in identifying the mechanisms underlying specific pathophysiological states [22]. Paus *et al*. found that stress can induce different parameters of skin immunology and hair biology in C57BL/6 and CBA/J mice [23]. In addition, basal and ultraviolet radiation B (UVB) stimulated differential expression of the cutaneous HPA axis in C57BL/6 and DBA/2J strains [24]. Thus, we expect different mouse strains to have distinct central and peripheral responses reflecting stress system activity.

This study aimed to explore the different effects of exposure to chronic restraint stress on hair follicle function and involvement of the central HPA axis among different mouse strains. We used a 21-day chronic restraint stress (CRS) protocol with five mouse strains commonly used in many research areas, including inbred C57BL/6, CBA/J, C3H/HeN, BALB/c and outbred ICR mice to mimic clinical heterogeneity. Using behavioral assessments, we selected C57BL/6, CBA/J and BALB/c mice, all of which were inbred strains and had significant alterations in all behavioral parameters. Then, we examined the effects of stress on hair follicle growth and melanogenesis in skin system. Additionally, we compared the different responses of the central HPA axis with regard to gene and protein levels. Our findings may provide a better understanding of the brain-skin connection and assist in systematically discovering and screening potent pharmacological agents for psychocutaneous disorders.

## Materials and methods

### Animals

Five to six-week-old, syngenic, male C57BL/6, CBA/J, C3H/HeN, BALB/c and ICR mice (weighing 18–22 g) were obtained from Better Biotechnology Company (China). The animals were group-housed in standard conditions (12h light/dark cycle; lights on 06:00–18:00; temperature of 21–24°C; humidity 50–60%) at Experimental Animal Center of Southeast University in China, and all were provided with sterilized water and food *ad libitum*. All animals were left undisturbed and allowed to acclimatize to the conditions for 7 days prior to the onset of the experiment. Care and use of the animals were in accordance with protocols approved by the Animal Study Committee of China Pharmaceutical University (Permit Number: PMY33069N).

### Application of stress

All mice of each strain were randomly divided into two groups: the control group (ctrl) and chronic restraint stress group (crs) (n = 10 each group). For the CRS, 50 ml conical tubes with fifty to sixty spaced 3 mm-diameter holes for ventilation were used to apply the restraint stress on mice. Mice were placed head first into tubes with enough space for limited movements. The mice were not physically compressed and did not experience any pain. The duration of restraint stress lasted for 6 hours daily (10:00 a.m.-16:00 p.m.) [25]. The stress procedure lasted for 21 days before the behavioral testing began, and mice in the control group were kept in their original cage. Neither food nor water were provided during the period of stress in all groups.

### Anagen induction

**Fig 1:**
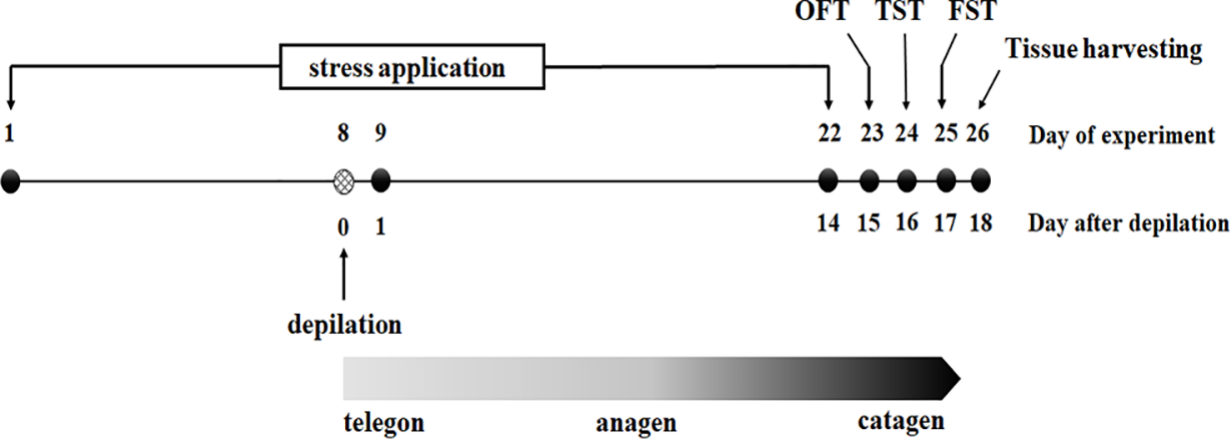
Time table of the experiments. CRS was applied on day 1 and lasted for 21 days. The depilation of all mice was on day 8 of the experiment to induce anagen (The day on depilation did not apply restraint stress). Behavioral tests (OFT, TST, FST) were examined on days 23, 24, 25 and tissue harvesting was collected on day 26 of the experiment.

Anagen was induced by depilation on day 8 after the experiment began (Fig 1). A wax/rosin mixture was applied to the dorsal skin of the mice with all hair follicles in telogen; peeling-off the wax/rosin mixture removed all hair shafts and immediately induced homogeneous anagen development [26]. Restraint stress was not applied on the day of depilation.

### Open-field test (OFT)

The open-field test was used to detect locomotor activity and rearing frequencies on day 23 after the experiment began. The open field, which was made of black wood (30×30×20 cm), was divided into 25 (6×6 cm) identical sectors by white stripes. The mice were placed in the center, and observation of their behavior began immediately and continued for 3 min. The locomotor activity (number of squares crossed) and rearing frequencies of the mice were measured by observers who were blind to the treatment conditions. The apparatus was cleaned in the interval between each test.

### Tail suspension test (TST)

The tail suspension test was conducted according to the method of Steru *et al*. on day 24 after the experiment began [27]. Mice were acoustically and visually isolated and then suspended on the edge of a shelf 50 cm above the floor by adhesive tape placed approximately 1 cm from the tip of the tail. The total duration of immobility was quantified during a 6-min test. Mice were considered immobile only when they hung passively and remained completely motionless.

### Forced Swim Test (FST)

The forced swim test was carried out according to the method of Porsolt *et al*. on day 25 after the experiment began [28]. Mice were placed individually into open cylindrical containers (height 25 cm, diameter 15 cm) containing 15 cm of water at 25 ± 1°C. Immobility during the last 4 min of the 6 min testing period was measured. Mice were considered immobile when they floated in the water without struggling.

### Tissue preparation

On day 26 after the experiment began (day 18 after depilation), all mice were sacrificed by cervical dislocation under deep anesthesia. Blood samples were collected by an eyeball removal method in less than one minute after mice were dead and centrifuged at 3000 rpm for 10 min to obtain serum, which was stored at -70°C immediately. Skin and brain samples of all mice were harvested. Parts of the skin samples were fixed in 4% paraformaldehyde for hematoxylin and eosin (H&E) staining. The remaining samples were stored in liquid nitrogen for western blot analysis and quantitative PCR.

### Histological analysis and quantitative histomorphometry

Serial paraffin sections (4-μm thick) were cut and stained with hematoxylin and eosin. All sections were analyzed at ×100 and ×400 magnification under an Olympus BX41 fluorescence microscope. The total number of hair follicles was quantified in 10 visual fields per mouse for 3 mice each group [29].

## Western blot analysis

Murine skin and brain protein samples were lysed in cold lysis buffer (0.1 M phosphate-buffered saline, pH 6.8, 1% Triton X-100, 1 mM phenylmethyl sulfoxide, and 0.01% aprotinin). Samples containing 30 μg of protein each were separated by sodium dodecyl sulfate polyacrylamide gel electrophoresis, transferred onto nitrocellulose membranes, and then incubated with the appropriate antibodies overnight at 4°C. Blots were then incubated with peroxidase-conjugated secondary antibodies for 1 h at room temperature and visualized by using enhanced chemiluminescence (Intron, Daejeon, Korea). The following primary antibodies were used in this study: TYR (ab52493, Abcam, UK), TRP-1 (ab3312, Abcam, UK), TRP-2 (ab74073, Abcam, UK), POMC (sc20148, Santa Cruz, USA), CRF (sc10718, Santa Cruz, USA), GR (ab2768, Abcam, UK) and β-actin (A1978, Sigma, USA). Densitometric scanning of band intensities obtained from three separate experiments was used to quantify the change in protein expression (the control value was onefold in each case).

### Quantitative real-time PCR analysis

Total RNA was extracted from mouse dorsal skin using TRIzol (Invitrogen, USA) and first-strand cDNA synthesis was performed using Advantage RT-for-PCR (Takara, Japan). For conventional reverse transcription-polymerase chain reaction (RT-PCR) cDNA was amplified using Taq DNA polymerase (Takara, Japan). Comparable quantities of cDNA were ensured by amplification of GAPDH. Primers and cycling conditions for POMC, CRH, and β-actin were applied. Transcripts were all amplified by 40 cycles of the following: 95°C for 30 s (denaturation), 60°C for 30 s (annealing) and 72°C for 30 s (extension). Melting curve analyses were performed to confirm the absence of nonspecific bands. The expression levels of each gene were normalized against β-actin, then calculated as fold change using the 2^-ΔΔCT^ method and the results were obtained from at least three independent experiments according to the manufacturer’s protocols. Primer sequences are listed in Table 1.

**Table 1:** Primer sequences.

Genes	Species	Forward (F) and Reverse (R) primer sequences		Product size (bp)
POMC	mouse	F	TTGCTGAGAACGAGTCGGC	87
		R	GACCTGCTCCAAGCCTAATGG	
CRF	mouse	F	CTCACGTACTCCACCGACCG	130
		R	TGCCAAACCAGCACTTTTCA	
β-actin	mouse	F	CAGGTCATCACTATTGGCAACGAG	87
		R	GATGCCACAGGATTCCATACCC	

### Corticosterone analysis

Serum corticosterone concentrations were measured using an IBL-AMERICA Corticosterone rat/mouse ELISA kit (IBL, USA) according to the manufacturer’s instructions. Serum samples were incubated at room temperature and then directly used for detection.

### Statistical analysis

All data were represented as the mean ± SD. Statistical analysis of results was performed using unpaired, two-tailed Student’s t test or one-way ANOVA with Tukey’s correction for multiple comparisons. All data were analyzed using GraphPad Prism software (UK). P<0.05 was considered to indicate significance.

## Results

### Effects of CRS on body weight and spleen index among five mouse strains

**Fig 2:**
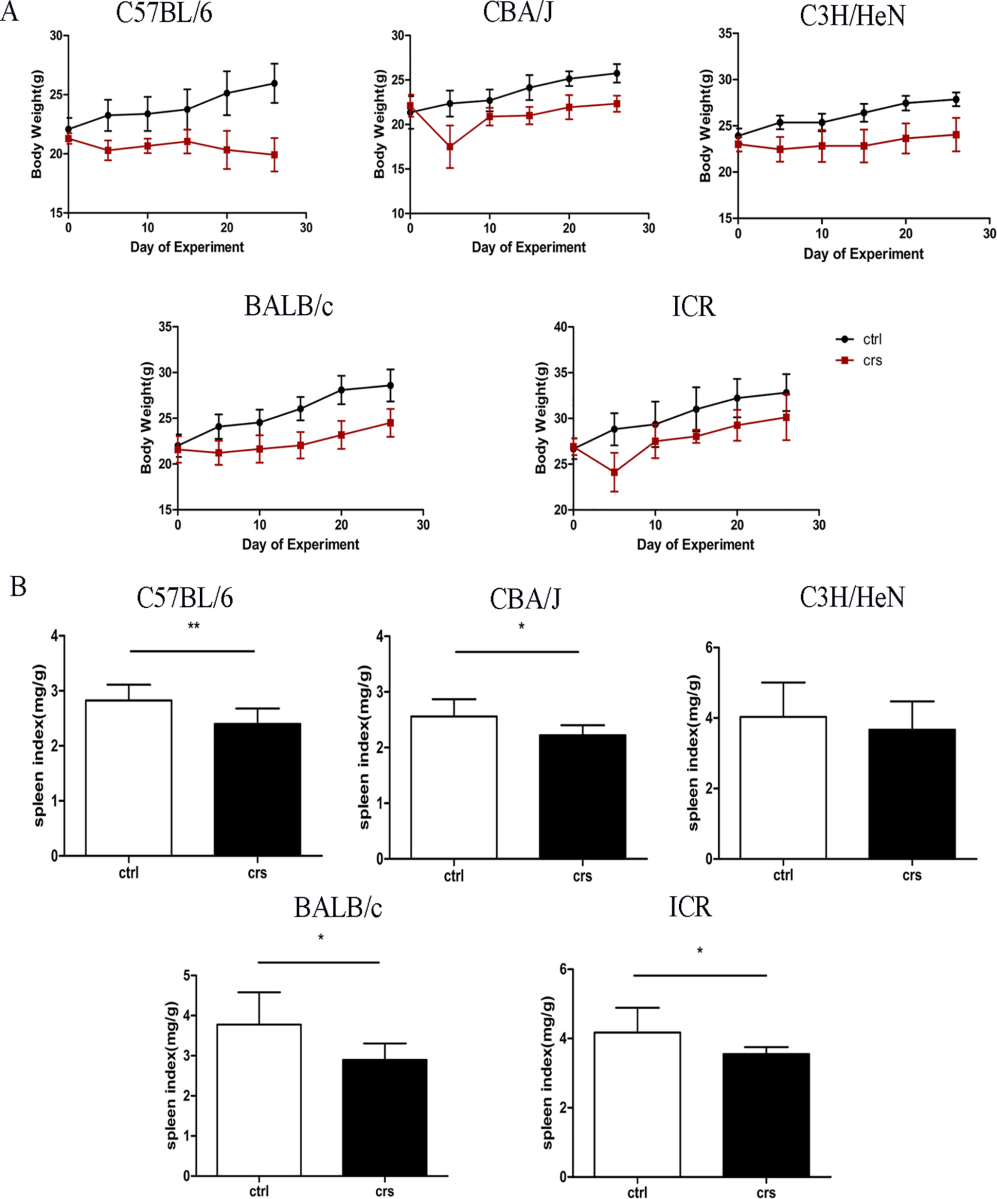
Effects of CRS on body weight and spleen index among different mouse strains. (A) On days 5, 10, 15, 20 and 25 after the experiment began, body weight was inhibited in CRS group among five mouse strains. (B) Effect of CRS on spleen index among five mouse strains. Data were showed as means ± SD, n = 8–9 for each group. *p<0.05, **p<0.01 compared with the control group.

To confirm whether CRS successfully induced psychological stress in five mouse strains, body weight was measured on days 5, 10, 15, 20 and 25 after the experiment began. As shown in Fig 2A, CRS inhibited the body weight gain of five mouse strains on days 5, 10, 15, 20 and 25 compared with their control groups. In addition, the spleen index was examined. The results depicted in Fig 2B show that CRS caused a significant reduction in spleen index in C57BL/6, CBA/J, BALB/c and ICR mice compared with their control groups (p<0.05, p<0.01).

### Effects of CRS on behavioral parameters among five mouse strains

To evaluate anxiety-like behaviors involved in the stress reaction, the open-field test, tail suspension test and forced swim test were applied.

**Fig 3:**
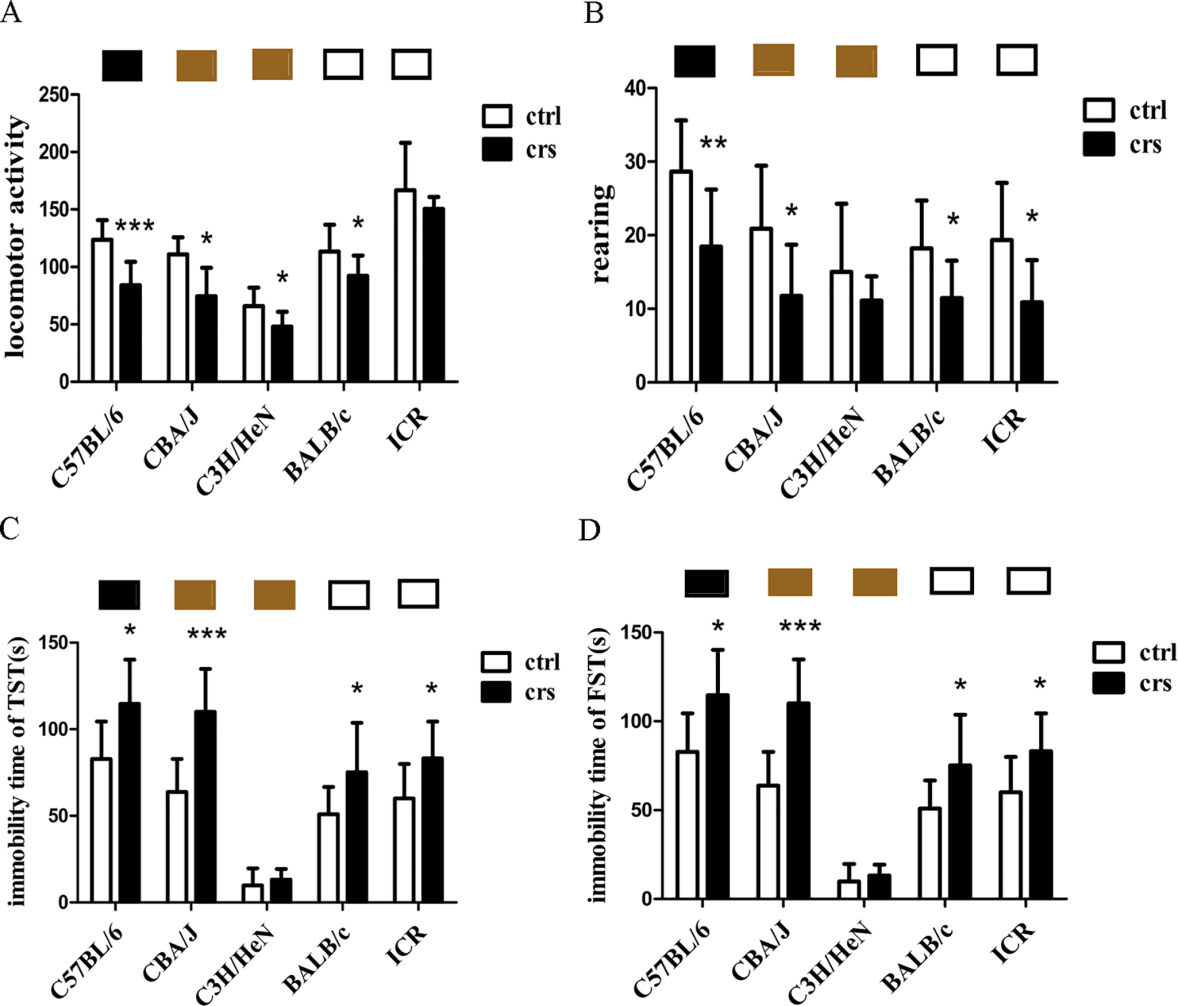
Effects of chronic restraint stress on behavioral parameters among different mouse strains. Effects of CRS on locomotor activity (A) and rearing (B) among different mouse strains in the open-field test. (C) Effect of CRS on immobility time among different mouse strains in the tail suspension test. (D) Effect of CRS on immobility time among different mouse strains in the forced swimming test. n = 8–9 for each group. *p<0.05, **p<0.01, ***p<0.001 compared with the control group.

In the open-field test, CRS significantly reduced locomotor activity in C57BL/6, CBA/J, C3H/HeN and BALB/c mice compared with their control groups (p<0.001, p<0.05) (Fig 3A). In addition, CRS significantly reduced the number of rearing events in C57BL/6, CBA/J, BALB/c mice and ICR mice compared with their control groups (p<0.01, p<0.05) (Fig 3B). Among these five strains, we observed the greatest differences in locomotor activity (p<0.001) and number of rearing events (p<0.01) in C57BL/6 mice between the control and stressed groups (Fig 3A and 3B).

The effect of CRS on immobility time in the tail suspension test shown in Fig 3C. CRS significantly increased immobility time in C57BL/6, CBA/J, BALB/c and ICR mice compared with their control groups (p<0.05, p<0.001). CBA/J mice showed the greatest differences (p<0.001) between the control and the stressed groups among the five strains.

The immobility time in the forced swim test shown in Fig 3D. CRS significantly increased the immobility time in C57BL/6, CBA/J, C3H/HeN and BALB/c mice (p<0.05, p<0.01, p<0.001). C57BL/6 mice showed the largest difference (p<0.001) between the control and stressed groups among the five strains.

Above all, the present results show a clear difference in responses among mouse strains under exposure to CRS. Through the comparative tests of behavior, we chose three inbred strains including C57BL/6, CBA/J and BALB/c mice for further study because of their significant behavioral changes under stressful condition compared with their respective control groups.

### Effects of CRS on hair follicle growth in C57BL/6, CBA/J, and BALB/c mice

**Fig 4:**
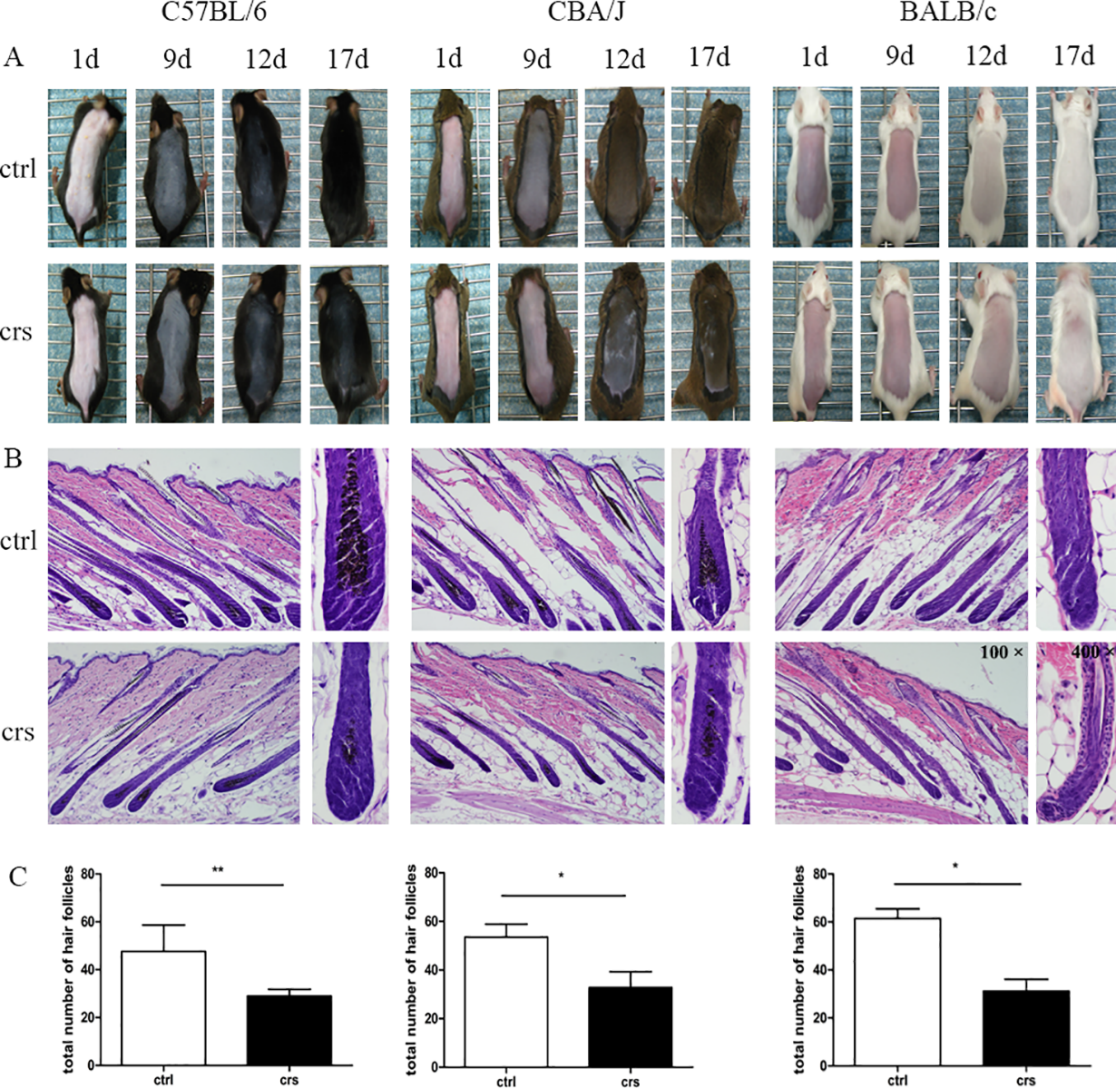
Effects of CRS on hair follicle growth in C57BL/6, CBA/J, and BALB/c mice. (A) Daily observation of mouse dorsal skin on days 1, 9, 12, 17 after depilation. (B) Effect of CRS on hair follicular morphology (Magnificance: 200×, 400×). (C) Effect of CRS on total number of hair follicles among three mouse strains. * p<0.05, **p<0.01 compared with the control group.

To investigative the effects of CRS on hair growth among the three selected strains, murine back skin color was observed daily, and photographs were taken. On days 12 and 17 after depilation (Fig 4A), stressed mice of all three strains showed hair growth inhibition effects compared with their control groups.

In addition, H & E staining of the murine back skin sections in Fig 4B show the morphologic characteristics of murine hair follicles, including changes in dermal papilla shape and size and the content of melanocytes. According to the time-scale for hair cycle, the majority of hair follicles in stressed mice were in catagen II-III stage on days 12 and 17 after depilation, whereas the nonstressed mice were still in anagen VI. In the three control groups, we observed fully developed hair follicles with the bulb and dermal papilla located in the deep subcutis, whereas the hair follicles of three stressed groups showed narrow hair bulbs. In addition, there was a decrease in the quantity of melanin granules in the hair follicles of the stressed group compared with their control group in C57BL/6 and CBA/J mice.

To further study the inhibitory effects of CRS on hair follicles, we counted the total number of hair follicles of all three mouse strains. As shown in Fig 4C, CRS caused the number of follicles in C57BL/6, CBA/J and BALB/c mice to decrease significantly compared to their control groups (p<0.05, p<0.01). Above all, the hair follicle growth inhibition in C57BL/6 mice (p<0.01) was the most significant among the three strains.

### Effects of CRS on melanogenesis in C57BL/6, CBA/J, and BALB/c mice

**Fig 5:**
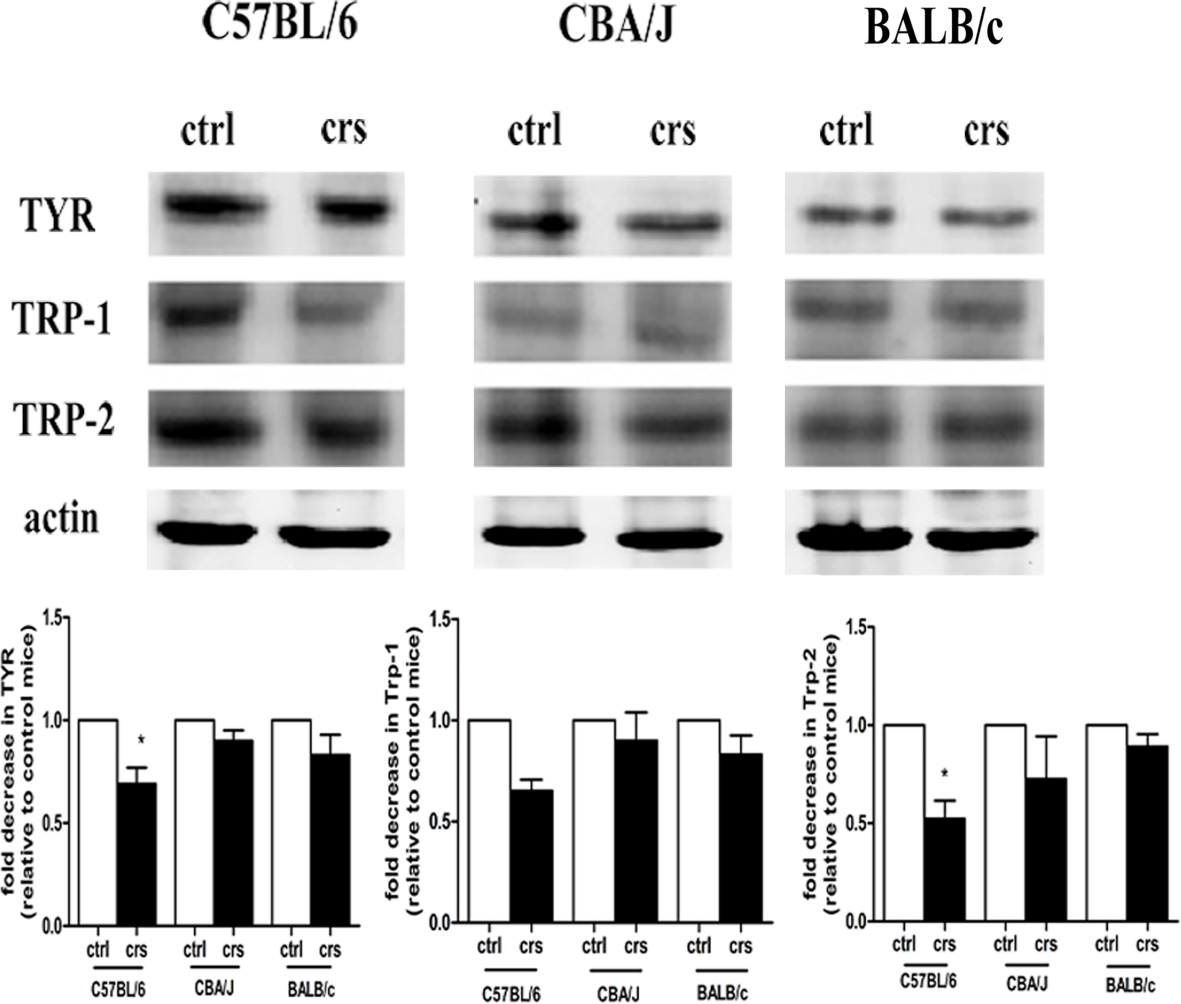
Effects of CRS on expression of TYR, TRP-1 and TRP-2 protein in C57BL/6, CBA/J, and BALB/c mice. Equal protein loadings were confirmed using an anti-β-actin antibody. Representative results from one of three experiments are shown. Densitometric scanning of band intensities obtained from three separate experiments was used to quantify the change of proteins expressions (control value taken as onefold in each case). Data showed the mean ± SD of three experiments (n = 9). *p < 0.05 compared with the control group.

To explore whether the involvement of the central HPA axis response to CRS could affect murine skin pigmentation and its possible molecular mechanism, the protein expression of key elements such as tyrosine (TYR), tyrosinase-related protein-1 (TRP-1) and tyrosinase-related protein-2 (TRP-2) protein were detected by western blot analysis. As shown in Fig 5, for C57BL/6 mice, significant decreased expression levels of TYR and TRP-2 were observed in the stressed group compared with the control group (p<0.05). However, there was no significant difference in TYR, TRP-1 and TRP-2 expression between the control and stressed groups in CBA/J and BALB/c mice (p>0.05).

Therefore, we suggest that CRS mostly inhibits melanogenesis in the back skin of C57BL/6 mice.

### Effects of CRS on the central HPA axis in C57BL/6, CBA/J, and BALB/c mice

**Fig 6:**
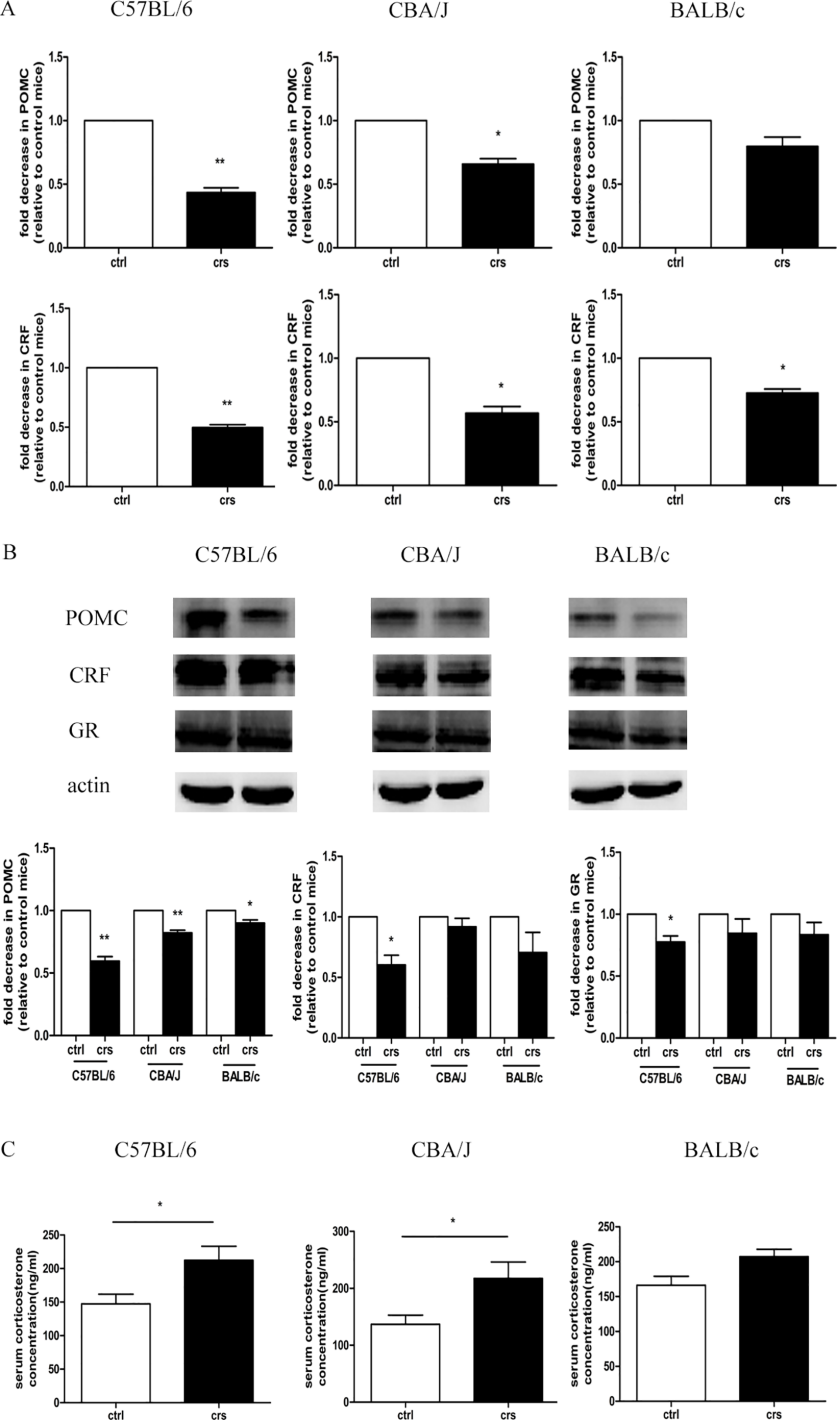
Effects of CRS on the central HPA axis in C57BL/6, CBA/J, and BALB/c mice. (A) The relative gene expression levels of the POMC and CRF was quantified by quantitative real-time PCR, n = 9. (B) Effect of CRS on expression levels of POMC, CRF and GR protein. Equal protein loadings were confirmed using an anti-β-actin antibody. Representative results from one of three experiments are shown. Densitometric scanning of band intensities obtained from three separate experiments was used to quantify the change of proteins expressions (control value taken as onefold in each case), n = 9. (C) Effect of chronic restraint stress on serum corticosterone level. n = 6. Data showed the mean ± SD of three experiments. *p < 0.05, **p<0.01 compared with the control group.

To investigative the effects of CRS on the central HPA axis among the three strains, we used quantitative real-time PCR to detect the expression levels of POMC and CRH mRNA in the murine brain. As shown in Fig 6A, CRS significantly decreased the expression levels of POMC and CRF mRNA in stressed C57BL/6 mice compared to control group (p<0.01). Similar to the results for C57BL/6 mice, POMC and CRF mRNA levels in stressed CBA/J mice were also decreased compared to the control group (p<0.05). However, there was no significant difference in the expression of POMC mRNA in BALB/c mice (p>0.05).

In addition, the protein expression of key elements in the central HPA axis, including POMC, CRF and GR, were detected in the murine brain (Fig 6B). We found that CRS significantly decreased the expression of POMC protein in all three strains compared to their control groups (p<0.05, p<0.01). Compared to the control group, CRS significantly reduced the protein expression levels of CRF and GR in C57BL/6 mice (p<0.05). This decreased effect was observed in CBA/J and BALB/c mice, but our results were not significant (p>0.05).

To further examine the central HPA axis responses in the process, we used an ELISA method to examine the concentration of serum corticosterone (CORT). As shown in Fig 6C, CRS significantly increased the level of serum corticosterone in C57BL/6 and CBA/J mice (p<0.05). There was no significant difference between the control and stressed groups in BALB/c mice (p>0.05).

Thus, we suggest that the central HPA axis was mostly affected by CRS in C57BL/6 mice compared with the other two strains.

### Homology analysis of POMC, CRH and NR3C1 protein sequence in C57BL/6, CBA/J, and BALB/c mice

**Fig 7:**
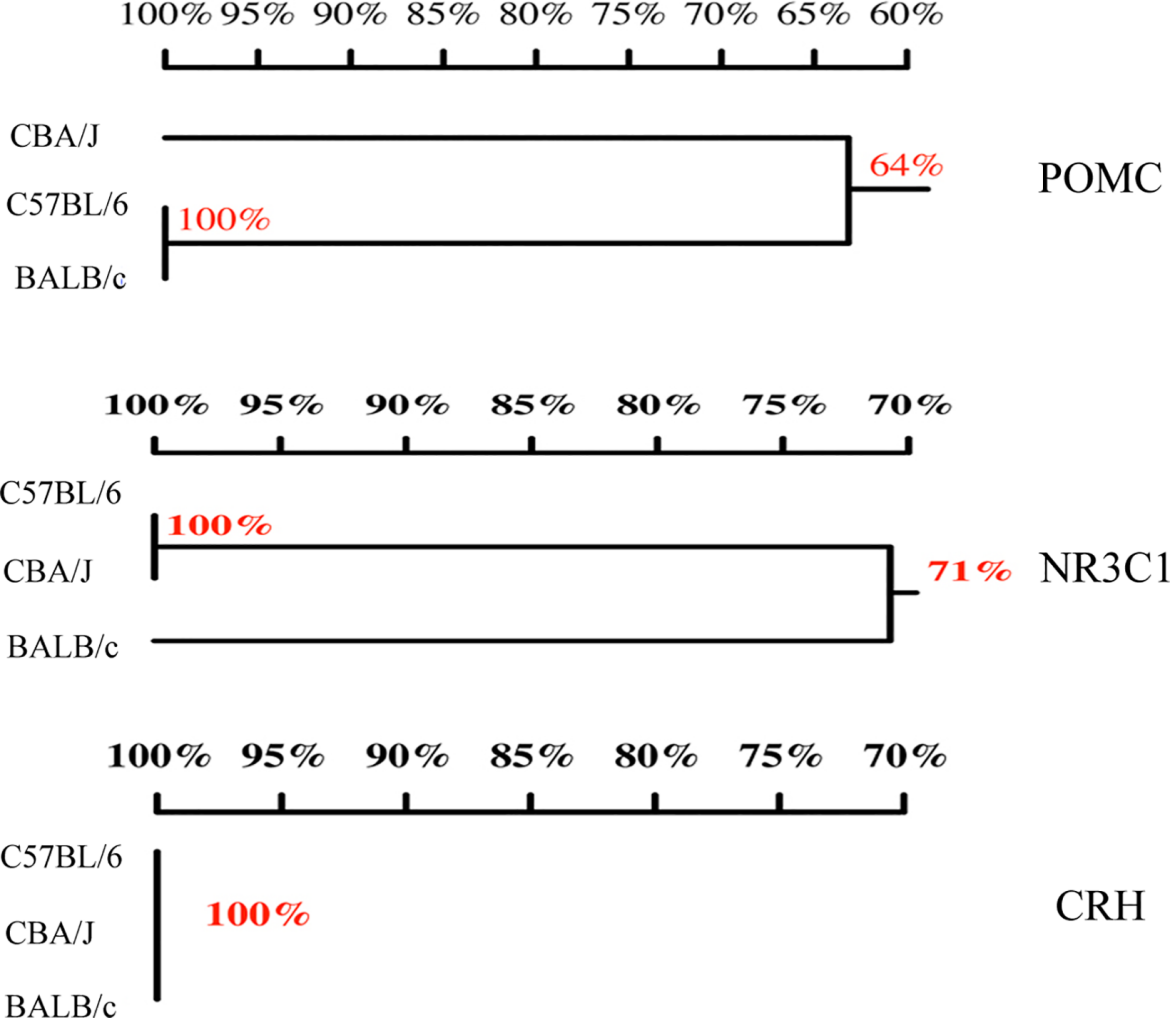
Homology analysis of POMC, CRH and NR3C1 protein sequence in C57BL/6, CBA/J, and BALB/c mice.

The protein homology analysis results revealed that the POMC sequence of CBA/J mice is 64% identical to that of C57BL/6 and BALB/c mice, and the glucocorticoid receptor (NR3C1) sequence of BALB/c mice is 71% identical to that of C57BL/6 and CBA/J mice. There are no differences in the CRH sequences among the three mouse strains (Fig 7).

### A summary of the impact of CRS on the central HPA axis and hair follicle function among the three mouse strains

Table 2 summarizes the impact of CRS on the central HPA axis and hair follicle function in C57BL/6, CBA/J and BALB/c mice. CRS mostly affected the central HPA axis of C57BL/6 mice by inhibiting the expression of POMC, CRF, and GR and increasing the concentration of serum CORT. For hair follicle function, CRS inhibited hair growth and melanogenesis in C57BL/6 mice with greater significance compared to the other two strains.

**Table 2:** A summary of the impact of CRS on the central HPA axis and hair follicle function among the three mouse strains.

	Strains			
	C57BL/6	CBA/J	BALB/c	
Behavioral test				
Locomotor activity (OFT)	***	*	*	
Rearing (OFT)	**	*	*	
Immobility time (TST)	*	***	*	
Immobility time (FST)	***	*	**	
Responses in hair follicle function				
Total number of hair follicles	**	*		*
TYR protein (melanogenesis)	*	ns		ns
TRP-1 protein (melanogenesis)	ns	ns		ns
TRP-2 protein (melanogenesis)	*	ns		ns
Responses in central HPA axis				
POMC mRNA	**	*	ns	
CRF mRNA	**	*	*	
POMC protein	**	*	*	
CRF protein	**	ns	ns	
GR protein	*	ns	ns	
CORT concentration	*	*	ns	

Data are expressed as the mean ± SD.

*p < 0.05

**p<0.01 and

***p<0.001 compared with the control group. ns = no significance.

## Discussion

In this study, we selected three inbred mouse strains including C57BL/6, CBA/J and BALB/c mice from five initial mouse strains to detect and compare the different effects of a 21-day exposure to chronic restraint stress (CRS) on hair follicle function and the central HPA axis. We found that CRS may inhibit hair follicle growth and melanogenesis though regulating the key elements of central HPA axis. C57BL/6 mice had the most significant reaction to the stress application with regard to hair follicles and the central HPA axis, indicating that the C57BL/6 strain is responsive to 21-day chronic restraint stress.

We employed a 21-day chronic restraint stress model to induce emotional effects on mice without causing biological injury [30]. This stress procedure mimics the depressive situation and induces symptoms resembling those of human patients [31]. In our research, CRS caused delayed weight gain in all strains, implying that CRS is sufficient to induce a stressful state [32]. Furthermore, the application of CRS procedures resulted in a significantly decrease in spleen index among the four strains (C57BL/6, CBA/J, BALB/c and ICR). Previous studies have identified that CRS indeed influences the immune system, they found that the spleen contributes to stress-induced blood leukocyte changes [33,34].

After exposure to CRS, we detected the psychological status among the five mouse strains. Behavioral performance was examined using the open-field test, tail suspension test and forced swim test, all of which have been widely used for assessing anxiety and depression of animal in stressful situation [35]. The locomotor and rearing activities in the open-field test reflect the natural extinction of orientation and exploratory behavior [36,37]. Prolonged immobility time in the tail suspension test and forced swim test reveals a failure of persistence in escape-directed behavior [38]. Above all, the present results showed that all mouse strains developed some degree of depression and that there were considerable behavioral differences across the strains used under exposure to CRS. These differences may be due to adaptations of the central HPA axis. C57BL/6, CBA/J and BALB/c mice showed significant changes between their control and stressed groups in all behavioral indexes, and C57BL/6 mice displayed a much higher decrease in locomotor activity, climbing response and immobility time than the other two strains in the open-field test and forced swim test. These results are partly consistent with a study by Valeria Carola *et al*., who observed that non-agouti mice with dark fur exhibited decreased locomotion and more aggressive-like behavior compared to agouti mice with light fur [39].

From the comparative behavioral results, we selected three strains including C57BL/6, CBA/J and BALB/c mice for further study on hair follicle and central HPA axis function. These three strains were chosen based upon significant behavioral changes under stressful condition compared with their control groups. In addition, they are all inbred strains because of their multiplication of a unique individual, whereas outbred strains are characterized by a considerable genetic variability [40,41].

To evaluate the effects of CRS on hair follicle function, we examined hair follicle growth and melanogenesis among three selected strains. Our results showed degenerative morphology and reduced numbers of hair follicles in three stressed groups. For further quantitative analysis, our data showed that C57BL/6 mice exhibited a larger difference in the decreased number of hair follicles than the other two strains. Hair follicle pigmentation appears to be regulated by mediators involved in the neuroendocrine stress response [8,42,43]. In our study of pigment activity, we examined the key elements of factors such as TYR, TRP-1 and TRP-2 protein in melanogenesis [44]. The results of western blots showed the expression levels of TYR and TRP-2 in the stressed C57BL/6 group were decreased significantly compared with the control group. However, there was no significance of TYR, TRP-1 and TRP-2 expressions between control and stressed group in CBA/J and BALB/c mice. Therefore, we suggest CRS mostly inhibited hair follicle growth and melanogenesis of C57BL/6 mice among these three strains.

Stress induced different responses in hair follicle growth and melanogenesis among different mouse strains, and we are interested in modulation by psychological means. To date, there are few studies on the regulation of central HPA axis on skin function. To further explore the different responses in the central HPA axis among the three mouse strains, we examined the mRNA and protein expressions of key elements in the central HPA axis.

POMC, CRF and GR are the key players in the central HPA axis and participate in stress response [45]. Detecting alterations in their mRNA and protein levels may provide insight into the neurochemical mechanisms. Our results showed that CRS most significantly reduced the expression levels of POMC and CRF mRNA and POMC, CRF, and GR protein in C57BL/6 mice, indicating that the function of central HPA axis was more inhibited in C57BL/6 mice in the other two strains. As the final product released from adenohypophysis, the level of blood cortisol reflects the HPA axis response to stress [46–48]. In rodents, corticosterone is the primary stress hormone [49]. The ELISA result revealed that the concentration of serum corticosterone in C57BL/6 and CBA/J mice was significantly increased after stress stimuli, whereas BALB/c mice showed no significant difference in corticosterone level. The significant increase in serum corticosterone eventually induced hair follicle dysfunction. As a stress hormone, cortisol can change and modulate hair follicle biology and the hair cycle [50,51]. It has been proved that high cortisol concentrations can reduce the synthesis and accelerate the degradation of important skin elements, such as hyaluronan and proteoglycans by approximately 40% [52]. Hair cortisol concentrations in the human scalp have a positive correlation with urine or serum cortisol produced by the central HPA axis. Zhang *et al*. observed corticosterone was produced in significantly greater quantities in alopecia areata-mice than in normal mice only submitted to repeated restraint stress but not in case of acute exposure to stressful events [53]. In addition, studies have reported that the plasma level of cortisol in alopecia areata-patients is higher than that in healthy controls [54]. In summary, three mouse strains showed inhibited expressions of POMC, CRF and GR mRNA and protein in response to CRS, and the central HPA axis function was suppressed significantly in C57BL/6 mice. However, contradictory results have been reported indicating that BALB/c mice are more susceptible to stress than C57BL/6 mice after exposure to foot shock and 2 h acute stress, while we report a contrasting result here. These inconsistent responses may due to distinct type, duration, frequency of stress and the timing of sampling. Acute stress may be related to hyperreactivity in the HPA axis, whereas hyporeactivity may occur in the HPA axis under chronic stress [55]. Exposure to different stressors seems to be important for the final response [48].

Above all, we found that different effects on hair follicle function after stress stimuli are based on differential modulation of the central HPA axis. Our previous results on behavior and central HPA axis were strain-dependent, indicating differences in the genetic background. Based on sequences acquired from the database at GenBank (http://www.ncbi.nlm.nih.gov/genbank), we performed a protein sequence homology analysis of POMC, CRH and the glucocorticoid receptor gene (NR3C1) among C57BL/6, CBA/J and BALB/c mice. These three genes are associated a number of nervous system disorders and play an important role in the stress-induced central HPA axis response [56]. We found different genotype homology among different mouse strains, indicating that the genetic background may alter central HPA axis function. Considering the involvement of the central HPA axis in nervous-skin system modulation, these differences in gene profile key elements may lead to different responses after exposure to stress. Previous studies have reported that genetic factors may contribute to the pharmacological sensitivity to antidepressive agents [57–59]. Therefore, the selection of an appropriate animal strain underlines the importance in experiments and clinical research.

In this research, we found that 21-day chronic restraint stress may inhibit hair follicle growth and melanogenesis through regulation of the key components in the central HPA axis. Stress stimuli suppressed central HPA axis activity via decreasing the mRNA and protein expression levels of CRF, POMC and GR in mice. Eventually the altered central HPA axis activity may be involved in modulating hair follicle function in the skin system. The mechanism needs to be studied further and may be mediated through complex bidirectional cooperation between the nervous, endocrine and immune systems. In addition, the interaction between the central HPA axis and peripheral HPA axis should be explored and elucidated. Our study demonstrated that C57BL/6, CBA/J and BALB/c mice display significant differences in behavioral assessments between their control and stressed groups. The results revealed that different mouse strains differ in their sensitivity to 21-day chronic restraint stress and exhibit different responses in the central HPA axis. C57BL/6 is the most sensitive strain among the three strains tested. The depressed function in the central HPA axis induced by stress may be correlated with hair follicle function.

At present, our research still has limitations as we only examine the most important targets and receptors in the central HPA axis. For further study, we will investigate the expression of more gene and protein to find more targets related to the central nervous system and skin. In addition, C57BL/6 strain may provide an ideal animal model to study the possible pathophysiological mechanisms by which the nervous system influences skin function and to screen dermatological drugs suitable for psychotherapy. We believe the current study will provide some useful information for researchers who are interested in the bidirectional communication between the nervous and skin systems and the management of stress-induced cutaneous diseases.

## Supporting information

S1 File Protein sequences of POMC among C57BL/6, CBA/J and BALB/c mice. (PDF)

S2 File Protein sequences of NR3C1 among C57BL/6, CBA/J and BALB/c mice. (PDF)

S3 File Protein sequences of CRH among C57BL/6, CBA/J and BALB/c mice. (PDF)

S4 File Raw data set. (XLSX)
