# Supplementary material for: Involvement of the central hypothalamic-pituitary-adrenal axis in hair growth and melanogenesis among different mouse strains
Source: PLoS One. 2018 Oct 24;13(10):e0202955. doi: 10.1371/journal.pone.0202955 (PMC6200183; doi:10.1371/journal.pone.0202955)
Supplement: S1 File — (PDF) [file pone.0202955.s001.pdf]

```
7_CBA_J         GLHPGLQTRPLAGDARVSWQRRTAPDKPPEVRHGSLPLGPLRPQEQQQCWQRGAEACGGR
14_BALB_cJ      GLHPGLQTRPLAGDARVSWQRRTAPDKPPEVRHGSLPLGPLRPQEQQQCWQRGAEACGGR
20_C57BL_6J     GLHPGLQTRPLAGDARVSWQRRTAPDKPPEVRHGSLPLGPLRPQEQQQCWQRGAEACGGR

7_CBA_J         GGVGRWQFRAESTRGQALLLHGALPLGQAGGQETAPGEGVPQRCERVGGGLSPRVQEGAG
14_BALB_cJ      GGVGRWQSRAESTRGQALLLHGALPLGQAGGQETAPGEGVPQRCERVGGGLSPRVQEGAG
20_C57BL_6J     GGVGRWQSRAESTRGQALLLHGALPLGQAGGQETAPGEGVPQRCERVGGGLSPRVQEGAG

7_CBA_J         RRAAIRLGAGPGVRRGEGRRALPGGALPLEQPAQGQALRWLHDLREEPDAPGDALQERHH
14_BALB_cJ      RRAAIRLGAGPGVRRGEGRRALPGGALPLEQPAQGQALRWLHDLREEPDAPGDALQERHH
20_C57BL_6J     RRAAIRLGAGPGVRRGEGRRALPGGALPLEQPAQGQALRWLHDLREEPDAPGDALQERHH

7_CBA_J         QERAQEGPVRVQGSFHSKAPSLHGRADDLPLRVTCVRKNLSDFTVGSDLQKTAIKSKHNC
14_BALB_cJ      QERAQEGPVRVQGSSHSKAPSLHGRADDLPLRVTCVRKNLSDFTVGSDLQK.LRKS.QNT
20_C57BL_6J     QERAQEGPVRVQGSSHSKAPSLHGRADDLPLRVTCVRKNLSDFTVGSDLQK.LRKS.QNT

7_CBA_J         PVTLGVPPILFASAETRPDTWKMPRFCYSRSGALLLALLLQTSIDVWSWCLESSQCQDLT
14_BALB_cJ      TVQLHG..SLQSCLPLQRLGLTRGRCRDSATVAQGPCCWPSCFRPPMCGAGAWRAASART
20_C57BL_6J     TVQLHG..SLQSCLPLQRLGLTRGRCRDSATVAQGPCCWPSCFRPPMCGAGAWRAASART

7_CBA_J         TESNLLEAGDTCWKDSGRESRVTINSWWSSFVVAVDVQTLVSLRILADLGWLHCDNYVGY
14_BALB_cJ      SPR.RATCWRQETEHVGKIAGEKAESQTPNGGVHLLLLTSKPSFLCASQIWGGCIVIITW
20_C57BL_6J     SPR.RATCWRQETEHVGKIAGEKAESQTPNGGVHLLLLTSKPSFLCASQIWGGCIVIITW

7_CBA_J         RTGRGTKREATEEKRGEQLREATEHLCPQRAAFPRQ
14_BALB_cJ      VIGQDGGPNGRRRKRKEVKSSDERPLNIFVPRELPFRDR
20_C57BL_6J     VIGQDGGPNGRRRKRKEVKSSDERPLNIFVPRELPFRDR
```
