# Supplementary material for: Involvement of the central hypothalamic-pituitary-adrenal axis in hair growth and melanogenesis among different mouse strains
Source: PLoS One. 2018 Oct 24;13(10):e0202955. doi: 10.1371/journal.pone.0202955 (PMC6200183; doi:10.1371/journal.pone.0202955)
Supplement: S2 File — (PDF) [file pone.0202955.s002.pdf]

```
20_C57BL_6J    VVENLLSYCFQTFLDKSMSIEFPEMLAEIITNQIPKYSNGNIKKLLFHQKLPYERLPRKL
14_BALB_cJ     VVENLLSYCFQTFLDKSMSIEFPEMLAEIITNQIPKYSNGNIKKLLFHQKLPYERLPRKL
7_CBA_J        VVENLLSYCFQTFLDKSMSIEFPEMLAEIITNQIPKYSNGNIKKLLFHQKLPYERLPRKL

20_C57BL_6J    NLLLLYKLINLSCRCFVVLFVCLVCFLYALHVVSRGPRLGNRSRAITFQQESRQCALAGV
14_BALB_cJ     NLLLLYKLINLSCRCFVVLFVCLVCFLYALHVVSRGPRLGNRSRAITFQQESRQCALAGV
7_CBA_J        NLLLLYKLINLSCRCFVVLFVCLVCFLYALHVVSRGPRLGNRSRAITFQQESRQCALAGV

20_C57BL_6J    SQKLEQLVETCPLSEKDRTTTSAQSPCDQLSAQLSVGWITLSRLFCWCIFPMYSDSILIY
14_BALB_cJ     SQKLEQLVETCPLSEKDRTTTSAQSPCDQLSAQLSVGWITLSRLFCWCIFPMYSDSILIY
7_CBA_J        SQKLEQLVETCPLSEKDRTTTSAQSPCDQLSAQLSVGWITLSRLFCWCIFPMYSDSILIY

20_C57BL_6J    AWKPEKSLHVYIRKGKLCLLLLLSGFNNFLYIQTMLARFSIIFVF.LLYSCLRWAAASQL
14_BALB_cJ     AWKPEKSLHVYIRKGKLCLLLLLSGFNNFLYIQTMLARFSIIFVFQLLYSCLRWAAASQL
7_CBA_J        AWKPEKSLHVYIRKGKLCLLLLLSGFNNFLYIQTMLARFSIIFVFQLLYSCLRWAAASQL

20_C57BL_6J    STLTLISVKWVGAPTLMALSYQKTTEIDSDLQYSCQKLLLCIYLLPWGIIVVQINRPNWY
14_BALB_cJ     STLTLISVKWVGAPTLMALSYQKTTEIDSDLQYSCQKLLLCIYLLPWGIIVVQINRPNWY
7_CBA_J        STLTLISVKWVGAPTLMALSYQKTTEIDSDLQYSCQKLLLCIYLLPWGIIVVQINRPNWY

20_C57BL_6J    RAPSPVTCWVNCGWLQKTNCKTVPTNRPRLHPMHHLFSGAIATKFVTQLFQDLREFVHFK
14_BALB_cJ     RAPSPVTCWVNCGWLQKTNCKTVPTNRPRLHPMHHLFSGAIATKFVTQLFQDLREFVHFK
7_CBA_J        RAPSPVTCWVNCGWLQKTNCKTVPTNRPRLHPMHHLFSGAIATKFVTQLFQDLREFVHFK

20_C57BL_6J    MYYSRPAVIKPRDCFNQTKLLSLNVTLNVSNMARMARHPFSHPTCHQLVLSWWYRKISYF
14_BALB_cJ     MYYSRPAVIKPRDCFNQTKLLSLNVTLNVSNMARMARHPFSHPTCHQLVLSWWYRKISYF
7_CBA_J        MYYSRPAVIKPRDCFNQTKLLSLNVTLNVSNMARMARHPFSHPTCHQLVLSWWYRKISYF

20_C57BL_6J    FVINMSGIHVCPTIHPYMCHRIHKSCELLHTESYHFKQNRSCSSPFCVHLTNFLLKAHTY
14_BALB_cJ     FVINMSGIHVCPTIHPYMCHRIHKSCELLHTESYHFKQNRSCSSPFCVHLTNFLLKAHTY
7_CBA_J        FVINMSGIHVCPTIHPYMCHRIHKSCELLHTESYHFKQNRSCSSPFCVHLTNFLLKAHTY

20_C57BL_6J    ATRNLISTGGQIICVIENKSNIKNMKLLIYFYIFFQIYIILVFTNLGREGLLQLYMQFIN
14_BALB_cJ     ATRNLISTGGQIICVIENKSNIKNMKLLIYFYIFFQIYIILVFTNLGREGLLQLYMQFIN
7_CBA_J        ATRNLISTGGQIICVIENKSNIKNMKLLIYFYIFFQIYIILVFTNLGREGLLQLYMQFIN

20_C57BL_6J    MIVKLYSVKEFLDEIVLSHVIYFLGSKKCWISYKIYSIEHPYRPQWSWKLKQVCSKLGRG
14_BALB_cJ     MIVKLYSVKEFLDEIVLSHVIYFLGSKKCWISYKIYSIEHPYRPQWSWKLKQVCSKLGRG
7_CBA_J        MIVKLYSVKEFLDEIVLSHVIYFLGSKKCWISYKIYSIEHPYRPQWSWKLKQVCSKLGRG

20_C57BL_6J    SWDWPCVQCRSGFDPIRSQGNFPPIPSSSDHGPVSAGWLYIQDRKVALMYLEAPSSNRVV
14_BALB_cJ     SWDWPCVQCRSGFDPIRSQGNFPPIPSSSDHGPVSAGWLYIQDRKVALMYLEAPSSNRVV
7_CBA_J        SWDWPCVQCRSGFDPIRSQGNFPPIPSSSDHGPVSAGWLYIQDRKVALMYLEAPSSNRVV

20_C57BL_6J    PTCSLRTGQKQRLKFGNNVHRGSSNMILKNRIGSQYTNPLHPTRVHFQVRGHVYVFFECF
14_BALB_cJ     PTCSLRTGQKQRLKFGNNVHRGSSNMILKNRIGSQYTNPLHPTRVHFQVRGHVYVFFECF
7_CBA_J        PTCSLRTGQKQRLKFGNNVHRGSSNMILKNRIGSQYTNPLHPTRVHFQVRGHVYVFFECF
```

```
20_C57BL_6J     LNVVIFSILQKLFNKKKYNHLLFEFSLKGNVQFVMVIGLKVLNNCNPAGCEIYGAEIPLE
14_BALB_cJ      LNVVIFSILQKLFNKKKYNHLLFEFSLKGNVQFVMVIGLKVLNNCNPAGCEIYGAEIPLE
7_CBA_J         LNVVIFSILQKLFNKKKYNHLLFEFSLKGNVQFVMVIGLKVLNNCNPAGCEIYGAEIPLE

20_C57BL_6J     DYQQCVSKASQKIIVHLWNVIWLNPKSTSHINLLSVPTSLAFKNRAPKKKR.KKKIYMLC
14_BALB_cJ      DYQQCVSKASQKIIVHLWNVIWLNPKSTSHINLLSVPTSLAFKNRAPKKKK.GKKRYICF
7_CBA_J         DYQQCVSKASQKIIVHLWNVIWLNPKSTSHINLLSVPTSLAFKNRAPKKKKRKKKIYMLC

20_C57BL_6J     YQKAADIHKTTIGSFPLDVRAILWYVGKKKAVIILIEYKERYGTVEQLLFCVWLHTVPNY
14_BALB_cJ      VINRRQQTFIKLLSEVFHMYKELSFGMWERRKLSFLSISERD.TVLFESSSFSACGFIPF
7_CBA_J         YQKAADIHKTTIGSFPLDVRAILWYVGKKKAVIILIEYKERYGTVEQLLFCVWLHTVPNY

20_C57BL_6J     VDFIIASVRIGNMPVLTTDLENYLLLVGQKGTFWLFALAWNTVYA..EERRQAIWGKEGD
14_BALB_cJ      QTMILLQELVTCLYDSQQILKTIFNYWDKKGHSGYFRHWLGTLYMQKKEDRQSGERKGTW
7_CBA_J         VDFIIASVRIGNMPVLTTDLENYLLLVGQKGTFWLFALAWNTVYA..EERRQAIWGKEGD

20_C57BL_6J     LGSTAFFKERHTNRDHPKGTGTTECESLVTSQVSSGELGEASPTSRAGTAGMGQAGTTIP    1
14_BALB_cJ      EALPSLRKDTPIDEIIPKAQGPQSVSPRVRALVSLEKPAPPAEQARQGWDKQGRQFQLDT    1
7_CBA_J         LGSTAFFKERHTNRDHPKGTGTTECESLVTSQVSSGELGEASPTSRAGTAGMGQAGTTIP    1

20_C57BL_6J     AGHWSQYFAPSYIPGSITVGTMVARFDLSALKDHGSLRELVLVGHPNDQLLNVFLMCLVS    1
14_BALB_cJ      GPSILLPLIYREAVSPWDEPWHVLICQHSRIMVAFGSFRFWLV.TPTISCSMCFLCAWFQ    1
7_CBA_J         AGHWSQYFAPSYIPGSITVGTMVARFDLSALKDHGSLRELVLVGHPNDQLLNVFLMCLVS    1

20_C57BL_6J     VLEGEIECAKDTANHFGWKFSHFPDYFRSAWSIKIGNQVFRKGLASIDMPERILFSDKWL    1
14_BALB_cJ      CKVKSVQRTLQTTSDG..SFLIFQTIFGQPGLSRSVTRSSGKGWLLSRTCLKGFYFLING    1
7_CBA_J         VLEGEIECAKDTANHFGWKFSHFPDYFRSAWSIKIGNQVFRKGLASIDMPERILFSDKWL    1

20_C57BL_6J     YENTLLNTLLNYIISVCQYSILYIKQMLYNGDKSILYCVWHYEAFSLFFITVIFKCVKIK    1
14_BALB_cJ      CMKIPSIPCLTTYRFQCVNILFCILNKCYIMGTNLYYTVYGIIKKLFHYFLSQFLNVKLK    1
7_CBA_J         YENTLLNTLLNYIISVCQYSILYIKQMLYNGDKSILYCVWHYEAFSLFFITVIFKCVKIK    1

20_C57BL_6J     N.QLLFKNKSCSFLFMLNN.LFKNLSFYYTVRCQTVKFCVEMFNFYFSFQFAVLVLPNHT    1
14_BALB_cJ      TSDSCLKIKVVVFYSCITCSLKTCLSTTQDVRLSFVWKCLT.FIFHFNLLFWYYQTT.HL    1
7_CBA_J         N.QLLFKNKSCSFLFMLNN.LFKNLSFYYTVRCQTVKFCVEMFNFYFSFQFAVLVLPNHT    1

20_C57BL_6J     FVMNWQMLVSHLQQCQIWINIIIKVSAFSLCDSQ                              1
14_BALB_cJ      IGSKCSAIYSNAKYGTSKYLLFHYVTP                                     1
7_CBA_J         FVMNWQMLVSHLQQCQIWINIIIKVSAFSLCDSQ                              1
```
