# Supplementary material for: Involvement of the central hypothalamic-pituitary-adrenal axis in hair growth and melanogenesis among different mouse strains
Source: PLoS One. 2018 Oct 24;13(10):e0202955. doi: 10.1371/journal.pone.0202955 (PMC6200183; doi:10.1371/journal.pone.0202955)
Supplement: S3 File — (PDF) [file pone.0202955.s003.pdf]

```
CBA_J        GLCTALCTEAIPGHQPFASGNTSNAMVLLHFFFFFSSVYW    40
BALB_cJ      GLCTALCTEAIPGHQPFASGNTSNAMVLLHFFFFFSSVYW    40
C57BL_6J     GLCTALCTEAIPGHQPFASGNTSNAMVLLHFFFFFSSVYW    40

CBA_J        QYTTFAVLLLFGEGRVFYCLGFFLKMKNKKLYIIYILYTD    80
BALB_cJ      QYTTFAVLLLFGEGRVFYCLGFFLKMKNKKLYIIYILYTD    80
C57BL_6J     QYTTFAVLLLFGEGRVFYCLGFFLKMKNKKLYIIYILYTD    80

CBA_J        THSHTDLMTRDSFSDQNQLAVKHCCLIHVMHKCIYVVKLF    120
BALB_cJ      THSHTDLMTRDSFSDQNQLAVKHCCLIHVMHKCIYVVKLF    120
C57BL_6J     THSHTDLMTRDSFSDQNQLAVKHCCLIHVMHKCIYVVKLF    120

CBA_J        ILFIKRYLKFYLCNMKEANHCNVFVLTSKKCKKKAIFTSP    160
BALB_cJ      ILFIKRYLKFYLCNMKEANHCNVFVLTSKKCKKKAIFTSP    160
C57BL_6J     ILFIKRYLKFYLCNMKEANHCNVFVLTSKKCKKKAIFTSP    160

CBA_J        TGPENFARGGAGGFPGSVVQSEKGQDSRQRGSQSQGRPIA    200
BALB_cJ      TGPENFARGGAGGFPGSVVQSEKGQDSRQRGSQSQGRPIA    200
C57BL_6J     TGPENFARGGAGGFPGSVVQSEKGQDSRQRGSQSQGRPIA    200

CBA_J        TAPGFACGHQVPGCLGPPSARAPQRAQIQRRQQEGLVQRL    240
BALB_cJ      TAPGFACGHQVPGCLGPPSARAPQRAQIQRRQQEGLVQRL    240
C57BL_6J     TAPGFACGHQVPGCLGPPSARAPQRAQIQRRQQEGLVQRL    240

CBA_J        LVPCAGPDRLHERSGMLVQLPLA                     263
BALB_cJ      LVPCAGPDRLHERSGMLVQLPLA                     263
C57BL_6J     LVPCAGPDRLHERSGMLVQLPLA                     263
```
